# Essential Oils as Natural Biocides in Conservation of Cultural Heritage

**DOI:** 10.3390/molecules25030730

**Published:** 2020-02-07

**Authors:** Franco Palla, Maurizio Bruno, Federica Mercurio, Antonella Tantillo, Valentina Rotolo

**Affiliations:** 1 Laboratory of Biology and Biotechnology for Cultural Heritage, Department of Biological, Chemical, and Pharmaceutical Science and Technologies, University of Palermo, Via Archirafi 38, 90123 Palermo, Italy; federica_mercurio@libero.it (F.M.); antotantillo86@gmail.com (A.T.); valentina.rotolo01@unipa.it (V.R.); 2 Laboratory of Natural Products, Department of Biological, Chemical, and Pharmaceutical Science and Technologies, University of Palermo, V. le delle Scienze Ed. 17, 90128 Palermo, Italy; maurizio.bruno@unipa.it

**Keywords:** biodeterioration, cultural assets, essential oils, GC–MS analysis, green conservation, insect repellent, microbial growth control

## Abstract

Essential oils (EOs) have been known for a long time, and they are used in several fields such as medicine and aromatherapy, as well as in the food and pharmaceutical industries. In the last decade, EOs have also been applied to contrast the biodeterioration of cultural heritage, representing a powerful resource in green conservation strategies. In this study, an integrated approach based on microscopic observation, in vitro culture, and molecular investigation was preliminarily employed to identify biological systems colonizing wooden artworks. In order to contrast the biodeterioration processes induced by fungal colonization (*Aspergillus flavus*) or insect infestation (*Anobium punctatum*), wooden artworks were exposed to the volatile compound of *Origanum vulgare* or *Thymus vulgaris* essential oils (EOs), the chemical composition of which was determined by GC–MS using both polar and apolar columns. Artwork exposure was performed in ad-hoc-assembled “clean chambers.” Evaluating the effects on biological systems, the compatibility with artwork constitutive materials, and the lack of negative effects on human health and environmental pollution, the use of EOs as a valid alternative to traditional biocides must be considered.

## 1. Introduction

The biodeterioration of organic cultural assets includes several biological systems able to induce complex alteration processes related to their biological and metabolic activity, artworks’ constitutive materials, and environmental conditions. Biological systems are able to accelerate some chemical and physical reactions, becoming detrimental for artwork conservation in both indoor and outdoor sites [1,2]. In order to inhibit or eradicate biological colonization, several chemical procedures—sometimes in combination with physical methods—are usually utilized. Common active components include benzalkonium chloride, permethrin, sodium fluoride, or other molecules applied for the disinfection of fungi and insects. These chemical compounds are generally toxic and not degradable, being persistent in the environment and causing uncontrollable contamination in areas far from the site of application [3,4].

Over the last decades, trends in biodeterioration control have indicated the need for biocide procedures using non-harmful and non-toxic compounds whose efficiency is kept over time and without adverse effects on cultural heritage and human health. Particularly, due to their antimicrobial-repellent properties, well known since the ancient times [5], natural molecules such as plant essential oils (EOs) are applied for several purposes to contrast biological colonization [6,7,8], acting through different pathways such as the regulation of intermediary metabolism, activation or blocking of enzymatic reactions, direct effects on enzyme synthesis, or alteration of membrane structures [9,10]. The EOs derived from different vegetable matrices contain several secondary metabolites able to inhibit the growth of bacteria, yeasts, and molds. They act directly on the microbial cell by inhibiting its growth, inducing the deterioration of the cytoplasmic membrane, regulating intermediary metabolism, activating or inhibiting enzymatic reactions, or affecting the enzyme synthesis [11,12,13,14].

Recently, EOs have been applied in conservation strategies, highlighting their antimicrobial activity against fungi and bacteria associated with the biodeterioration of cultural assets [15,16,17,18].

Our preliminary study showed that *Origanum vulgare* and *Thymus vulgaris* EOs had strong antimicrobial activity in in vitro assays, which has been successfully confirmed by in situ application on the complex biofilm revealed under the floor mosaic tesserae in the Greco-Roman archeological site of Solunto, Sicily (Italy). the antimicrobial activity of 15% *T. vulgaris* EO solution was enough to deeply impact the biofilm’s liveliness [19].

*O. vulgare*, belonging to the Lamiaceae family, typical in the Mediterranean basin, is an aromatic perennial plant growing spontaneously and cultivated all over the world. The plant is rich in carvacrol, a phenolic monoterpene with recognized anti-inflammatory and antitumor properties. *T. vulgaris* is also a plant of the Lamiaceae family (about 300 evergreen taxa, native to Asia, North Africa, and Southern Europe) and has active aromatic molecules with strong antiseptic and antibacterial properties. It contains phenolic monoterpenes such as thymol (30%–70%) and carvacrol (3%–15%).

**Figure 1:**
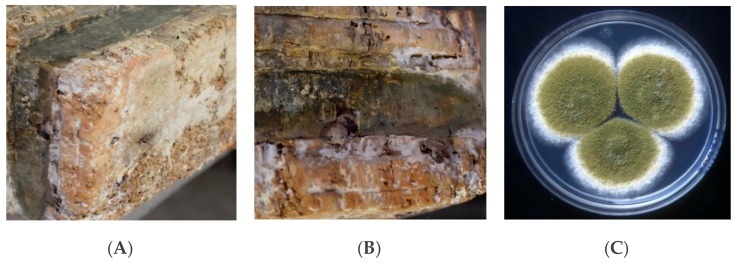
Base of a wooden sculpture (BWS): (**A**,**B**) noticeable fungal colonization; (**C**) *Aspergillus flavus* colonies isolated on Sabouraud agar.

**Figure 2:**
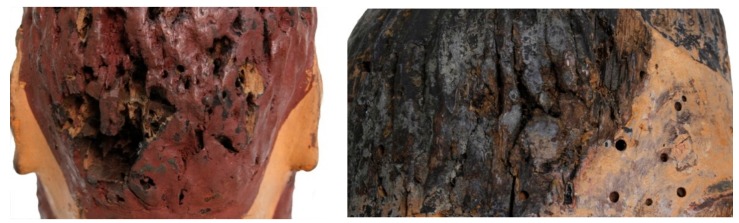
Wooden puppet heads (WPHs): deep deterioration of constitutive materials (**left**); flight holes 1.5–3 mm in diameter (**right**) related to the *Anobium punctatum* (de Geer 1774) infestation.

In this study, the activity of *Origanum vulgare* and *Thymus vulgaris* EOs were investigated as a green biocide against *Aspergillus flavus* colonies isolated from the base of a wooden sculpture (Figure 1A,B) or as a green repellent of *Anobium punctatum* insects that had infested the heads of wooden Sicilian puppets (Figure 2); both artifacts are from the 18th century.

The application was performed in ad-hoc-constructed “clean chambers” in order to expose the artifacts to the volatile compounds of EOs for 15–20 days.

The results allow us to consider the use of EOs as a valid alternative to commercial pesticides by providing a prospective application in the field of the green conservation of Cultural Heritage.

## 2. Results

Combining microscopy analysis, in vitro culture, and molecular biology investigation [19,20], a diffused *Aspergillus flavus* colonization was identified on the base of a wooden sculpture (BWS), as shown in Figure 1, while *A. punctatum* (Anobiidae family) infestation was revealed in wooden puppet heads (WPHs), as seen in Figure 2. The infestation represents a grave risk to the conservation of wooden artifacts, since those insects can digest cellulose substrates and several generations can re-infest the same wooden structure.

**Figure 3:**
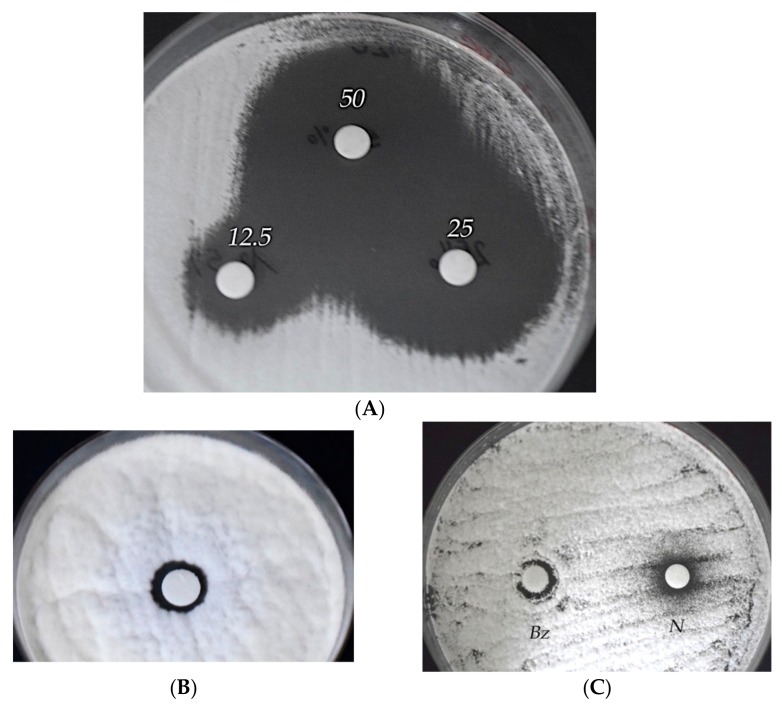
Agar disc diffusion method. Evaluation of the antimicrobial activity vs. *Aspergillus flavus* of: (**A**) *Thymus vulgaris* essential oil (EO), 12.5%, 25%, and 50% solutions; (**B**) 70% Et–OH; (**C**) 0.2% *v/v* benzalkonium chloride (Bz) or Nipagin-M (N).

In order to set up green conservation strategies, the antimicrobial activity of *O. vulgare* and *T. vulgaris* EOs were in vitro assayed against *Aspergillus flavus* colonies by the agar disc diffusion method [21]. As shown in Figure 3, the growth-inhibition-halo diameters underline the good antimicrobial activity of both EOs, as shown in Figure 3A for the *T. vulgaris* EO, Et–OH (Figure 3B), and benzalkonium chloride and Nipagin-M commercial biocides (Figure 3C).

The average diameters of the inhibition halos, measuring the antimicrobial action (sensible ≥ 6 mm, resistant < 6 mm), are reported in Table 1.

Considering the repellent activity and use in insect-pest management [22], the potential effect of these EOs on *A. punctatum*, infesting four WPHs, was also evaluated.

**Figure 4:**
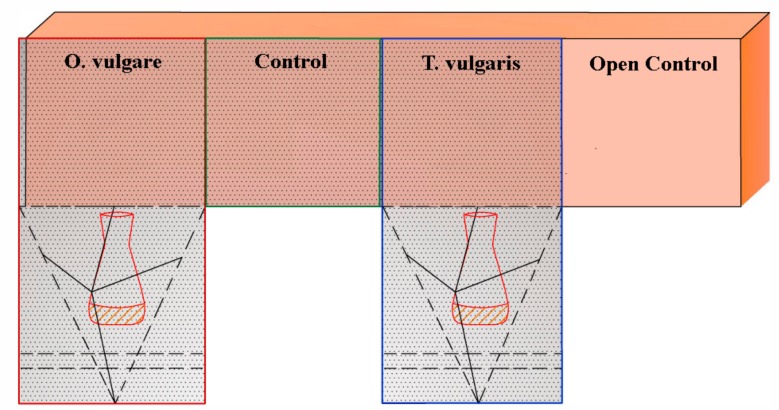
BWS surface. The ad-hoc-assembled structure (clean chamber) allowed the exposure of the *A. flavus* colonized areas to *O. vulgare* or *T. vulgaris* EOs volatile compounds (50%, solutions dispensed in each open sterile glass container). Two controls were performed: C = low-oxygen concentration (clean chamber without an EO); OC (Open Control) = *A. flavus* colonized area, maintained in the original environmental conditions.

**Figure 5:**
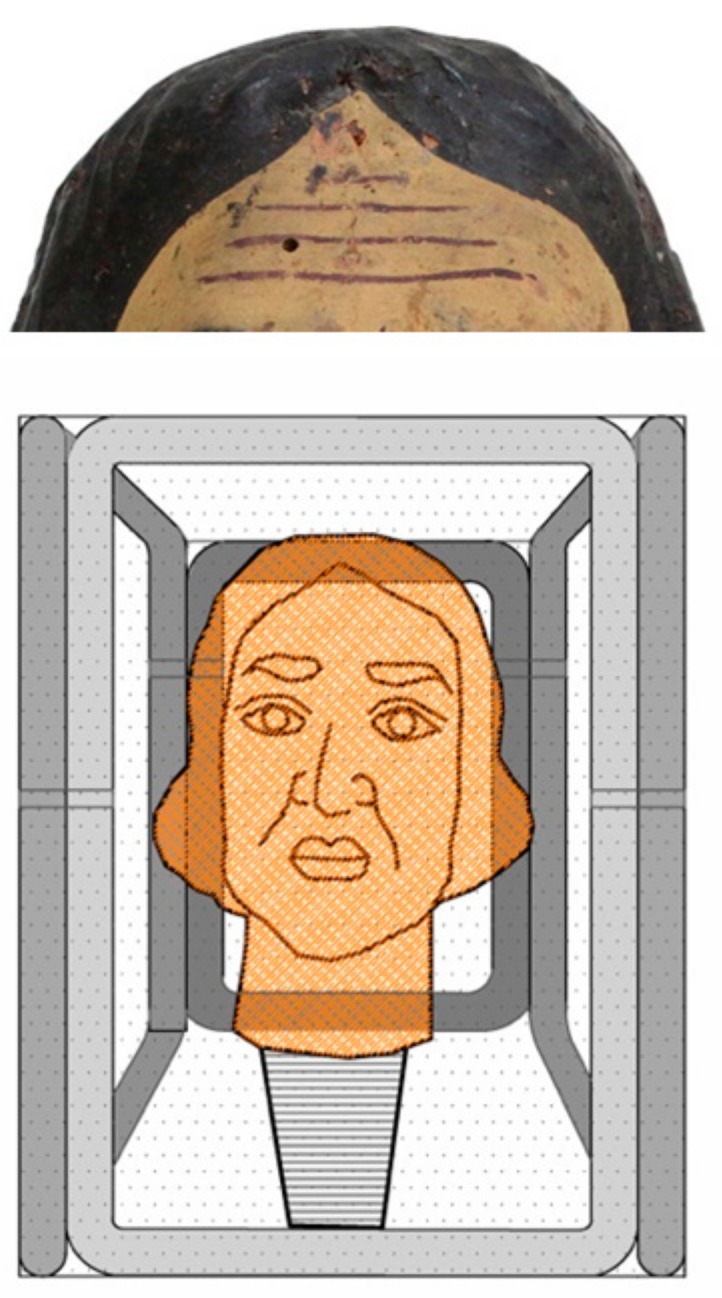
The ad-hoc-assembled structure for the treatment of *A. punctatum*-infested WPHs with the *O. vulgare* or *T. vulgaris* EO volatile compounds. Each head was centered in a cubic close-packing; 50% EO solutions were dispensed in a pierced glass container.

Wooden artifacts were exposed to volatile compounds of the EOs, assembling ad hoc structures (clean chambers) as shown in Figure 4 for the BWS and in Figure 5 for the WPHs respectively. 

These structures allowed the combination of EO volatile compound exposition in low-oxygen environments.

**Figure 6:**
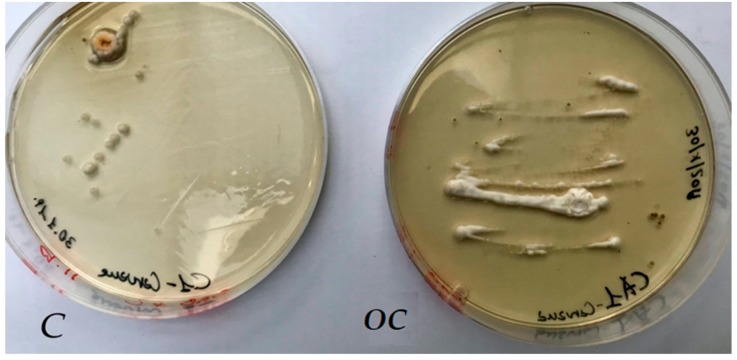
*A. flavus* colony grew on Sabouraud agar plate inoculated with samples collected from Control (C) and Open Control (OC) areas; any colony was observed from samples collected in the two BWS areas exposed to *O. vulgare* or *T. vulgaris* EO volatile compounds, respectively.

After the treatment of the BWS, the sampling of the exposed to EO volatile compounds, Control (C) and Open Control (OC) areas, was repeated by sterile cotton swabs, followed by inoculation of fresh Sabouraud agar media. Several *A. flavus* colonies were observed only for Control and Open Control samples (Figure 6), instead, any fungal colony grew for EO exposed samples.

The chemical composition of *T. vulgaris* and *O. vulgare* EOs was analysed by GC–MS and the main components (>1%) are listed in Table 2 according to their retention indices on an HP 5MS column.

The *T. vulgaris* EO is particularly rich in phenolic compounds (carvacrol 64.96% and thymol 8.25%) with a good quantity of their biogenetic precursor *p*-cymene (11.29%). According to previous literature [23], several chemotypes of thyme, based on essential oil compositions, have been established, including (1) linalool, (2) borneol, (3) geraniol, (4) sabinene hydrate, (5) thymol, and (6) carvacrol, as well as a number of multiple-component chemotypes. It has been reported [24,25] that the majority of commercial *T. vulgaris* essential oils are derived from thymol chemotypes, but according to our analysis, we suggest that the oil used in the present work belongs to the carvacrol chemotype.

*O. vulgare* is an extremely variable species, which is reflected in a plethora of scientific names that have been given to the presently recognized *O. vulgare* and to other kinds of variation in the *O. vulgare* complex. The populations of Southern Europe are quite rich in volatiles, whereas those growing in Central and Northern Europe are considered to be poor sources of essential oils. Individual plants rich in essential oils (2% or more) usually accumulate large amounts of phenolic monoterpenes deriving from the “cymyl”-pathway (mainly carvacrol and/or thymol and their biosynthetic precursors γ-terpinene and p-cymene). According to the proportions of cymyl compounds, sabinyl compounds, and the acyclic linalool/linalyl acetate, three different main monoterpene chemotypes were defined. The cymyl and acyclic pathways were usually active in plants from the Mediterranean climate whereas an active sabinyl pathway was characteristic of plants from the Continental climate [26]. As reported in Table 1, the *O. vulgaris* EO is quite rich in thymol (27.18%) and *p*-cymene (11.29%), clearly showing its belonging to the cymyl chemotypes.

## 3. Discussion

Biological systems such as microorganisms and insects play a widely demonstrated role in the biodeterioration of cultural assets [2,27]. In routine practice, pest colonization is countered by chemical pesticides, the toxicity and persistence of which are well known, and several factors such as toxicity to humans, risks of environmental pollution, and compatibility with substrates need to be discussed. Many biocides are difficult to degrade and are persistent in the environment, also causing contamination of the areas that are far from the site of treatment [28,29].

Natural products from vegetable matrices, such as EOs, contain a wide variety of secondary metabolites able to act against several biological systems and might be thought of as environmentally acceptable pesticides. Based on these peculiarities, conservation scientists have evaluated the use of some EOs to set up green procedures for cultural heritage protection. Researchers from different countries have tested the antimicrobial activity against microorganisms associated with biodeterioration in archives, libraries, and museums. Essential oils (lemon, spearmint, fennel, marjoram, and rosemary) at different concentrations have also been assayed against yeast colonies isolated from the Royal tombs (limestone and granite blocks) at Tanis [16,19,30,31,32,33,34,35]. The antimicrobial activity of *Ricinus communis* against bacteria isolated from indoor air in the document repositories of the National Archives of Cuba has been determined [36]. Considerable interest in the *Origanum vulgare* EO has been established in agricultural, pharmaceutical, and cosmetic industries [37]. The antifungal activity of the *Origanum vulgare, Rosmarinus officinalis*, and *Lavandula angustifolia* (Lamiaceae) EOs has been investigated against fungi such as *Bipolaris spicifera* and *Epicoccum nigrum* and *Aspergillus niger*, *Aspergillus ochraceus*, *Trichoderma viride*, and *Penicillium sp*., isolated from stone and wooden objects, respectively [31]. The results allow us to hypothesize new applicative protocols for the protection of cultural heritage, based on EOs directly applied to biofilm [38] or as volatile compounds presented here. In this case, it should also be investigated if the exposure occurs in low-oxygen-concentration microenvironments that probably contribute to the biocidal effect of EOs [39]. The antifungal activity of *Origanum vulgare* and *Thymus vulgaris* has been evaluated by Lavin and colleagues against *Scopulariopsis sp*. and *Fusarium sp*., isolated from paper documents [40]. Particularly, the good antimicrobial activity of the *Thymus* EO against *Bacillus subtilis* and *Staphylococcus epidermidis* (comparable to chloramphenicol and ketoconazole), as well as against *Fusarium oxysporum* and *Aspergillus niger* (frequently infesting archives, libraries, and historical art craft objects), has been demonstrated [18]. Furthermore, plant essential oils can be applied in the prevention of fungal infections, demonstrating an antifungal activity greater than some commercial fungicides, which due to their selective action and safety can be applied in sustainable conservation protocols [41,42].

Even today, the use of natural products is widespread in the pharmaceutical, sanitary, cosmetic, agricultural, and food industries. Drugs derived from plants are effective, easily available, less expensive, and rarely have side effects, and their use in cultural asset conservation can surely contribute to human and environmental health, following the modern restoration procedures. Furthermore, equally as cheap is the manufacturing of “clean chambers,” as described in this study, and there are opportunities to use them in health conditions and well-attended cultural heritage environments (exhibition halls, museums, libraries, etc.).

While the monitoring of these artifacts is still ongoing, it is encouraging to not observe any fungal or insect re-colonization after eight months post-treatment.

## 4. Conclusions

In the present study, the potential use of the *Origanum vulgare* and *Thymus vulgaris* EOs in the field of cultural heritage preservation is clearly shown. Particularly, wooden artworks were exposed to their volatile compounds in order to contrast fungal colonization or insect infestation.

The antimicrobial property of the EOs was preliminarily tested in vitro through the agar diffusion disc and microdilution methods. The variation in size of the inhibition halo between the EOs, Et–OH, benzalkonium chloride, and Nipagin-M solutions was probably due to the variability in the cell wall, the protein and lipid composition of the cytoplasmic membrane, and specific physiological processes.

Evaluating the average inhibition halo diameter (mm) of both EOs, Et–OH, benzalkonium chloride, or Nipagin-M solutions, the agar disc diffusion assays showed quite strong antimicrobial activity of both EOs but a low activity for the other biocidal solutions vs. *Aspergillus flavus*.

This study confirmed the data reported in the previous literature about the difference in the antimicrobial activity of natural and commercial biocides, and for the first time showed the assembling of ad hoc structures for the exposition of wooden artifacts to the *O. vulgare* or *T. vulgaris* EO volatile compounds, avoiding any negative impact on the environment or operator health.

The chemical characterization was performed, the main components were defined, and these EOs were utilized according to modern bio-restoration procedures, adopting eco-sustainable approaches.

Although more studies are needed to evaluate their permanence and durability in relation to the thermo-hygrometric condition of storage/exposition environments, we consider that these natural pesticides could be used as a valid alternative in the control of the biodeterioration processes affecting cultural assets.

## 5. Materials and Methods

### 5.1. Essential Oils (EOs)

*Origanum vulgare* and *Thymus vulgaris* EOs (doTerra^®^) were utilized as 50%, 25%, and 12.5% solutions. EOs were obtained from the plant leaf by steam distillation and their main chemical components (>1%) of *Thymus vulgaris* and *Origanum vulgare* EOs were defined and described in Table 2.

### 5.2. Essential Oil Isolation and Chemical Characterization

The GC analysis was performed by the Agilent 7000C GC system (Agilent, Santa Clara, CA, USA), fitted with a fused silica Agilent HP-5MS capillary column (30 m × 0.25 mm i.d.; 0.25 µm film thickness), coupled to an Agilent triple quadrupole Mass Selective Detector MSD 5973; ionization voltage 70 eV; electron multiplier energy 2000 V; transfer line temperature 295 °C. Helium was the carrier gas (1 mL min^−1^) [43]. The other GC analysis was performed in a Shimadzu QP 2010 plus (Shimadzu, Kyoto, Japan) single-quadrupole GC–MS system, fitted with a Supelcowax 10 capillary column (30 m × 0.25 mm i.d.; 0.25 µm film thickness; Merck KGaA, Darmstadt, Germany); ionization voltage 70 eV; transfer line temperature 280 °C. Helium was the carrier gas (1 mL min^−1^). For both columns, the temperature was initially kept at 40 °C for 5 min, then gradually increased to 250 °C at a rate of 2 °C min^−1^, held for 15 min, and finally raised to 270 °C at 10 °C min^−1^. One microliter of diluted samples (1/100 *v/v*, in *n*-pentane) was injected at 250 °C automatically and in the spitless mode; transfer line temperature 295 °C.

### 5.3. Identification of Compounds

Identification of compounds was carried out using NIST 11, Wiley 9, FFNSC 2, and Adams [43] databases. These identifications were confirmed by linear retention indices (LRIs) with those available in the literature on the SciFinder database. Some of the compounds were also confirmed by the comparison of mass spectra and retention times with standard compounds available in the laboratory. The retention indices were determined in relation to a homologous series of *n*-alkanes (C8–C30) injected under the same operating conditions. Component relative (%) amounts were calculated based on GC peak areas without using correction factors.

### 5.4. Pests

#### 5.4.1. Fungal Colonization

Sterile cotton swabs were utilized to perform the sampling on the colonized base of a wooden sculpture and for the following inoculation of Sabouraud agar plate (dextrose agar + chloramphenicol); after incubation for 24/36 h at 30 °C, single fungal colonies were isolated. The characterization was carried out by a multidisciplinary approach based on microscopy observation, in vitro culture (Figure 1C), and molecular investigation [20], allowing the characterization of *Aspergillus flavus* colonies spread on the BWS surface. The molecular investigation was performed by analyzing the genomic DNA target sequences. Preliminarily, the isolated fungal colonies underwent five freezing (−80 °C) and thawing (+55 °C) cycles, in the presence of a 500 μL-1X TE buffer (10 mM Tris-HCl pH 8.0/1 mM EDTA). Genomic DNA was extracted by QI Amp DNA stool Kit (Qiagen), (modified by adding 5 mg/mL of Proteinase K (Invitrogen) and incubating at 65 °C for 4 h). The DNA was the template molecule in PCR reactions, utilizing the universal internal transcribed spacer primers, ITS1 (forward) and ITS4 (reverse), specific for fungi genomes. The amplification products (about 750 bp in length) were previously analyzed on 2.0% agarose gel (1X TBE, Tris-HCl/Borate/EDTA), staining the DNA molecules by Saber-safe gel stain (Invitrogen, USA). Aliquots of 50 ng were sequenced by Eurofins-Operon service and the similarity evaluated through the Blast platform referred to genomic databases (NIH, Bethesda, Maryland, USA) [44,45].

In order to define the minimum inhibitory concentration (MIC) and minimum fungicidal concentration (MFC), distinguishing between biocide or biostatic action, the micro-dilution method was performed [38]. In each well of a 96-well microtiter, 30 μL of the EO solutions (12.5%, 25%, 50%, and 100%) were added to a 30 μL liquid nutritive medium and an equal volume of microbial suspension. Benzalkonium chloride (0.2%, *v/v*) was utilized as Commercial reference Biocide. Microbial growth after 18 h of incubation at 30 °C was evaluated by estimating the optical density at 500–600 nm. The MIC value was measured as the lowest concentration corresponding to any visible microbial growth, after incubation at 30 °C. The MFC was determined as the lowest concentration of EO able to kill 99.5% of the original inoculum, evaluating on antimicrobial-free sub-culture [21].

#### 5.4.2. Insect Infestation

Analyzing by an optical microscope, the insect residues, coarse frass (rosura), and flight holes, 1.5–3 mm in diameter (Figure 2), the Coleoptera (*Anobium punctatum*, de Geer 1774) was identified.

### 5.5. Evaluation of Antimicrobial Activity

The EO antimicrobial activity vs. *Aspergillus flavus* was evaluated in vitro by the agar disc diffusion method. Specifically, the surface of Sabouraud agar (90-mm Petri dishes) was seeded by the fungal suspension (1 × 104 conidia/mL), then a paper disc (6 mm in diameter) wetted with 10 μL of each EO at different concentrations (12.5%, 25%, and 50%) was placed. Confluent microbial growth was observed after incubation at 30 °C for 24–36 h, except in the area surrounding the paper disc, where growth inhibition halos were distinguished. In Figure 3, the test of growth inhibition of the *A. flavus* colonies was performed by *T. vulgaris* (A), 70% ethanol (B), and 12.5% solutions of two commercial biocides (benzalkonium chloride and Nipagin) (C); each test was performed twice.

The average halo diameters (mm) are shown in Table 1 and sensitivity or resistance to EO antimicrobial activity was determined in relationship to the halo diameter (≥6 mm = sensible; <6 mm = resistant).

### 5.6. EO Volatile Compounds Exposition

Colonized/infested wooden artifacts were exposed to EOs in ad-hoc-assembled “clean chambers”—specifically, the base of a wooden sculpture (BWS) colonized by *A. flavus* fungal colonies, and wooden puppets heads (WPHs) infested by *A. punctatum* xylophagous beetles.

Artifacts were exposed to the volatile compounds of *O. vulgare* or *T. vulgaris*; 0.5 mL of EOs were dispensed in a glass container, in each “clean chamber” (volume of 1000 cm^3^), assembled using a gas-barrier thermo-sealed film, as shown in Figure 4,5.

Concerning the BWS, the fungal colonized surface was divided into four areas, distinguishing two exposed to EOs (clean chamber + *O. vulgare* or *T. vulgaris*) and two not exposed, tagged as Control (C = clean chamber without EO) or Open Control (OC = *A. flavus*-colonized area, maintained in the original environmental conditions). EOs were dispensed in open sterile glass containers (Figure 4). An Oregon Scientific datalogger (model JB913R) equipped with a microprobe was utilized to track In/Out-Min/Max Temp. and R.H. values.

For the *A. punctatum*-infested WPHs, the exposure to EO volatile compounds was carried out centering each head in a cubic close-packing (Figure 5). A Wee–Neckarsulm Temperature/Humidity Station (model HG00073A) equipped with a wireless device was utilized to monitor the thermo-hygrometric conditions.

The treatments were carried out in a dedicated room, continuously monitored, at Temp = 23 + 2 °C and R.U. = 58 + 2% values. After 20 days of treatment the “clean chambers” were carefully removed and the artifacts monitored again: the BWS areas were sampled by sterile swabs, utilized for inoculating Sabouraud agar plates, while in the WPHs, the presence of the *A. punctatum* infestation was checked using optical (Leica) and digital microscopes.

## Figures and Tables

**Table 1:** Antimicrobial activity vs. *Aspergillus flavus*.

Biocide	Conc. (%)	Inhibition Halo (mm)
*Origanum vulgare*	50.0	24
	25.0	84
	12.5	9
*Thymus vulgaris*	50.0	20
	25.0	14
	12.5	8
Ethanol	70	<6
Benzalk–Cl	0.2	<6
Nipagin M	0.2	<6

**Table 2:** Main components (>1%) of *Thymus vulgaris* and *Origanum vulgare* EOs.

LRI ^a^	LRI ^b^	Compound	*T. vulgaris* (%)	*O. vulgare* (%)	Id ^c^
927	1028	α-Pinene	1.49	1.75	1, 2, 3
943	1060	Camphene	1.03	-	1, 2, 3
992	1149	β-Myrcene	2.00	-	1, 2, 3
1029	1255	*p*-Cymene	11.29	18.97	1, 2, 3
1061	1248	γ-Terpinene	2.52	4.50	1, 2
1086	1534	β-Linalool	-	2.81	1, 2, 3
1195	1690	α-Terpieol	-	1.34	1, 2
1302	2174	Thymol	8.25	27.18	1, 2, 3
1305	2194	Carvacrol	64.96	4.04	1, 2, 3
1397	1579	β-Caryophyllene	-	5.89	1, 2, 3
1456	1653	α-Caryophyllene	1.40	3.61	1, 2
1584	2020	Caryophyllene oxide	-	2.00	1, 2, 3

^a^ Linear retention index on a HP-5 MS column; ^b^ linear retention index on a Supelcowax 10 capillary GC column; ^c^ 1: retention index; 2: MS, mass spectrum; 3: STD, standard.
